# Genome organization and evolution of a eukaryotic nicotinate co-inducible pathway

**DOI:** 10.1098/rsob.210099

**Published:** 2021-09-29

**Authors:** Eszter Bokor, Michel Flipphi, Sándor Kocsubé, Judit Ámon, Csaba Vágvölgyi, Claudio Scazzocchio, Zsuzsanna Hamari

**Affiliations:** ^1^ Department of Microbiology, University of Szeged Faculty of Science and Informatics, Szeged, Hungary; ^2^ Institute de Génétique et Microbiologie, Université Paris-Sud, Orsay, France; ^3^ Department of Microbiology, Imperial College, London, UK; ^4^ Université Paris-Saclay, CEA, CNRS, Institute for Integrative Biology of the Cell (I2BC), Gif-sur-Yvette 91198, France

**Keywords:** *Aspergillus nidulans*, ascomycetes, eukaryotic nicotinate utilization, gene cluster evolution, gene cluster co-regulation, horizontal gene transmission

## Abstract

In *Aspergillus nidulans* a regulon including 11 *hxn* genes (*hxnS*, *T*, *R*, *P*, *Y*, *Z*, *X*, *W*, *V*, *M* and *N*) is inducible by a nicotinate metabolic derivative, repressible by ammonium and under stringent control of the nitrogen-state-sensitive GATA factor AreA and the specific transcription factor HxnR. This is the first report in a eukaryote of the genomic organization of a possibly complete pathway of nicotinate utilization. In *A. nidulans* the regulon is organized in three distinct clusters, this organization is variable in the *Ascomycota*. In some *Pezizomycotina* species all 11 genes map in a single cluster; in others they map in two clusters. This variable organization sheds light on cluster evolution. Instances of gene duplication followed by or simultaneous with integration in the cluster, partial or total cluster loss, and horizontal gene transfer of several genes (including an example of whole cluster re-acquisition in *Aspergillus* of section *Flavi*) were detected, together with the incorporation in some clusters of genes not found in the *A. nidulans* co-regulated regulon, which underlie both the plasticity and the reticulate character of metabolic cluster evolution. This study provides a comprehensive phylogeny of six members of the cluster across representatives of all *Ascomycota* classes.

## Graphical abstract

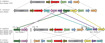

## 1. Introduction

Nicotinic acid (niacin, vitamin B3), a precursor of NAD and NADP, can be used by some bacteria as the sole nitrogen and carbon source. The common first step in all investigated prokaryotes is the hydroxylation of nicotinic acid (NA) to 6-hydroxynicotinic acid (6-NA). The further fate of 6-NA is variable; in *Pseudomonas* sp. it is converted to 2,5-dihydroxypyridine (2,5-DP) [1,2], in *Bacillus* sp*.* to 2,6-dihydroxynicotinic acid (2,6-NA) [3] and anaerobically to 1,4,5,6-tetrahydro-6-oxonicotinic acid in *Eubacterium barkeri* (formerly *Clostridium barkeri*) [4]. The detailed and variable further bacterial metabolic steps, whether aerobic or anaerobic, have been reviewed in [5].

The ascomycete fungus *Aspergillus nidulans* can use NA as its sole nitrogen source*.* In common with bacteria, a molybdenum cofactor (MOCO)-containing flavoprotein catalyses the conversion of NA to 6-NA (purine hydroxylase II, previously called xanthine dehydrogenase II, HxnS [6–9]). The *hxnS* gene is a paralogue of *hxA*, encoding a canonical xanthine dehydrogenase (HxA, purine hydroxylase I [10,11]) the latter being co-regulated with most other genes of the purine utilization pathway ([12,13] and references therein). The substrate specificities of HxA and HxnS have been studied in detail ([11] and references therein). In *A. nidulans* an NA-inducible co-regulated gene cluster is extant (hxn1/VI cluster, for cluster 1 in chromosome VI) comprising six genes, namely *hxnS*, *hxnR* (encoding the pathway-specific transcription factor), *hxnP* and *hxnZ* (encoding transporters of the major facilitator superfamily, which could play a role in the uptake of NA and/or NA-derivatives), and *hxnT* (putative flavin oxidoreductase) and *hxnY* (α-ketoglutarate-dependent dioxygenase) both which may be involved in the further metabolism of 6-NA [11]. In the 1970s, NA non-user mutants were isolated and genetically characterized [6]. These map in *hxnS* and *hxnR*, but also in a second gene cluster in chromosome VI (see below).

The hxn1/VI genes are specifically induced by a metabolite of NA catabolism but also expressed during nitrogen starvation [11] (RNASeq data [14] available at FungiDB, https://fungidb.org/fungidb/app). Expression of the *hxn* genes requires both the pathway-specific Zn-finger factor HxnR and the wide-domain GATA transcription factor AreA [11]. The latter mediates de-repression of a wide range of genes in the absence of preferred nitrogen sources (such as ammonium, l-glutamate and l-glutamine) [15–17]. The *hxnR* gene is defined by loss-of-function mutations which are non-inducible for the six genes of the cluster (including *hxnR* itself) and by constitutive mutations where transcription of all hxn1/VI genes occurs in the absence of inducer compounds [11]. The physiological involvement of the hxn1/VI cluster in nicotinate metabolism is further shown by the phenotype of null mutations in the *hxnR* gene, which result in the inability to use nicotinate, and two of its downstream metabolic derivatives as nitrogen sources [11].

Herein we complete the description of the genomic organization of the nicotinate-inducible *hxn* genes by the identification of five additional HxnR-dependent genes in *A. nidulans* and we describe variations in the genomic organization of the 11 *hxn* genes throughout the *Ascomycota* phylum.

The evolution of gene clustering in primary metabolism has been a subject of discussion. Specifically, we do not know which are the factors that lead to clustering of previously unclustered genes, those involved in clustering maintenance and those eventually leading to declustering [18]. Rokas and co-workers [19,20] have proposed that clustering confers a specific advantage when, in a given metabolic pathway, one or more intermediates are toxic, as single gene loss, leading to accumulation of a toxic metabolism, will be minimized. Notably, at least one toxic intermediate, 2,5-DP has been identified in the nicotinate degradation pathway [11], a compound that also occurs in prokaryotic pathways [1,2]. Investigating the diverse organization and evolution of the nicotinate regulon may contribute to this debate.

## 2. Results and discussion

### 2.1. Three HxnR-dependent, co-inducible gene clusters are extant in *Aspergillus nidulans*

**Figure 1.**
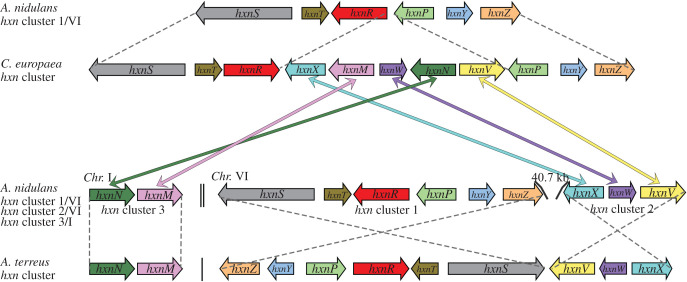
Expanded clusters in *Eurotiomycetes* uncover new *hxn* genes. Comparison of the organization of known [11] and putative novel *hxn* genes in three species: *A. nidulans*, *A. terreus* and *Cyphellophora europaea.* Each orthologous gene is symbolized by a thick arrow of a different colour, which also indicates relative orientation. Colour-coded double-headed arrows connect the five new putative *C. europaea hxn* genes to orthologues in the *A. nidulans* genome. Dashed lines connect similarly arranged cluster segments in the three species. For *A. nidulans,* a double vertical line indicates separation of clusters in different chromosomes (super-scaffold BN001306 for chromosome VI, BN001301 for chromosome I). For *A. terreus*, a single vertical line separates two distinct contigs (Contig AAJN01000215 for the nine-gene cluster, AAJN01000156 for the two-gene cluster). In *C. europaea*, the 11-gene cluster is contained in contig AOBU01000059.

In order to search for additional genes involved in nicotinate metabolism, we investigated the cluster structure in available ascomycete genomes (see below for a thorough description). Strikingly, in *Cyphellophora europaea* (*Pezizomycotina*, *Eurotiomycetes*, *Chaetothyriales*), five additional genes (to be called *hxnV*, *hxnW*, *hxnX*, *hxnM* and *hxnN*; see below) are positioned between *hxnP* and *hxnR* orthologues, forming a single, 11-gene cluster that includes all orthologues of the *A. nidulans hxnZ*, *hxnY*, *hxnP*, *hxnR*, *hxnT* and *hxnS* genes [11] (figure 1, *A. nidulans* cluster 1/VI; table 1). In *Aspergillus terreus* (and several other *Aspergillus* species; see below) *hxnV*, *hxnW* and *hxnX* are directly adjacent to *hxnS* (figure 1). In *A. nidulans* a cluster including *hxnX*, *hxnW* and *hxnV* (cluster 2/VI for cluster 2 in chromosome VI) is separated approximately 40 kb from *hxnZ* (deduced from the re-assembled genomic sequences [21]) while *hxnM* and *hxnN* are adjacent to each other in chromosome I (cluster 3/I for cluster 3 in chromosome I). While this article was being written, Martins *et al*. [22] suggested the clustered organization we described for *A. terreus* and *A. nidulans* and drew comparisons with a number of other species. However, these authors did not investigate the co-regulation by nicotinate or its metabolites of the putative new *hxn* genes.

**Table 1.** Results of *in silico* domain analysis of modelled Hxn proteins.

gene name, annotation no., protein length	corresponding cluster	cDNA accession number (NCBI)	name of identified domains^a^ (identification code, AA interval, *e*-value)	proposed enzyme class
HxnZ (AN11196) (533 AAs)	cluster 1/VI	MT707474 this work^b^	MFS1 (PF07690.13, 89–513 AAs, 4.0 × 10^−24^)	transporter
HxnY (AN11188) (349 AAs)	cluster 1/VI	MT707473 this work	PcbC (COG3491, 1–320 AAs, 3.85 × 10^−97^)/DIOX_N (PF14226, 7–131 AAs, 9.5 × 10^−30^)/2OG-FeII_Oxy (PF03171, 179–282 AAs, 5.8 × 10^−22^)	α-ketoglutarate-dependent dioxygenase
HxnP (AN11189) (491 AAs)	cluster 1/VI	KX585439 this work^b^	MFS1 (PF07690.13, 49–417 AAs, 3.2 × 10^−37^)	transporter
HxnR (AN11197)	cluster 1/VI	MT707475 this work	two C2H2 zinc finger domains (PF00096, 8–32 AAs and 41–63 AAs, 0.029 and 0.7)	transcription factor [11]
			fungal transcription specific domain (PF04082, 394–668 AAs, 2.0 × 10^−36^)	
			Amon *et al*. [11]	
HxnT (AN9177) (388 AAs)	cluster 1/VI	MT707472 this work	OYE-like FMN (cd02933, 9–368 AAs, 0 × 10^+00^);	old yellow enzyme
			FadH (COG1902, 6–387 AAs, 1.12 × 10^−117^)	
HxnS (AN9178) (1396 AAs)	cluster 1/VI	KX585438 Amon *et al*. [11]	Fer2 (PF14111, 14–82 AAs, 1.5 × 10^−06^)	xanthine dehydrogenase-type nicotinate dehydrogenase ([11] and refs therein)
			Fer2_2 (PF01799, 92–174 AAs, 3.8 × 10^−25^)	
			FAD_binding_5 (PF00941, 286–471 AAs, 8.9 × 10^−43^)	
			CO_deh_flav_C (PF03450, 480–586 AAs, 1.5 × 10^−30^)	
			Ald_Xan_dh_C2 (PF02738, 755–1296 AAs, 6.2 × 10^−203^)	
			Amon *et al*. [11] and refs therein	
HxnX (AN9161) (461 AAs)	cluster 2/VI	MN718567 this work	biH (COG0654, 17–414 AAs, 5.82 × 10^−44^)	FAD-dependent oxidoreductase
			FAD_binding_3 (PF01494, 16–235 AAs, 1.0 × 10^−09^);	
HxnW (AN11172) (254 AAs)	cluster 2/VI	MN718568 this work	adh_short_C2 (PF13561, 13–251 AAs, 1.2 × 10^−57^)	enoyl-(acyl carrier protein) reductase-like
HxnV (AN11187) (620 AAs)	cluster 2/VI	MN718569 this work^b^	PRK08294 (PRK08294, 7–620 AAs, 2.68 × 10^−93^)	phenol 2-monooxygenase-like enzyme
			FAD_binding_3 (PF01494, 23–380 AAs, 3.6 × 10^−76^)/UbiH (COG0654, 24–373 AAs, 9.70 × 10^−43^);	
			PHOX_C (cd02979, 435–616 AAs, 7.26 × 10^−18^)/Phe_hydrox_dim (PF07976, 404–574 AAs, 2.8 × 10^−26^)	
HxnN (AN10833) (543 AAs)	cluster 3/I	MN718565 this work	amidase (PF01425, 78–531 AAs, 8.5 × 10^−108^)	amidase
HxnM (AN6518) (307 AAs)	cluster 3/I	MN718566 this work	CE4_HpPgdA_like (cd10938, 8–287 AAs, 1. + 0 × 10^−133^)	C–N bond cleaving hydrolase-like
			CDA1 (COG0726, 43–145 AAs; 4.03 × 10^−21^)	

^a^Description of the abbreviated names of protein domains: MFS1, major facilitator superfamily; PcbC, isopenicillin N synthase and related dioxygenases; DIOX_N, non-haem dioxygenase in morphine synthesis N-terminal; 2OG-FeII_Oxy, 2OG-Fe(II) oxygenase superfamily; OYE-like FMN, old yellow enzyme (OYE)-like FMN-binding domain; FadH, 2,4-dienoyl-CoA reductase or related NADH-dependent reductase; Fer2 and Fer2_2, [2Fe-2S] binding domain; FAD_binding_5, FAD-binding domain; CO_deh_flav_C, CO dehydrogenase flavoprotein C-terminal domain; Ald_Xan_dh_C2, molybdopterin-binding domain of aldehyde dehydrogenase; UbiH, 2-polyprenyl-6-methoxyphenol hydroxylase and related FAD-dependent oxidoreductases; FAD_binding-3, FAD-binding domain; adh_short_C2, enoyl-(acyl carrier protein) reductase; PRK08163, salicylate hydroxylase; PRK08294, phenol 2-monooxygenase; PHOX_C, FAD-dependent phenol hydoxylase (PHOX) family, C-terminal TRX-fold domain; Phe_hydrox_dim, phenol hydroxylase, C-terminal dimerization domain; CE4_HpPgdA_like, catalytic domain of *Helicobacter pylori* peptidoglycan deacetylase (HpPgdA) (proposed as cyclic imidase) and similar proteins; CDA1, deacetylase, PgdA/CDA1 family.

^b^cDNA analysis revealed that automatic annotation was erroneous and the experimental determination of cDNA resulted in a corrected gene model.

The genomic organization of the *hxn* genes in *A. nidulans* chromosome VI confirms data obtained with a mutagenic screen, which yielded besides mutations in *hxnS* and *hxnR* [11] additional mutants unable to grow on either NA or 6-NA as sole nitrogen sources. A number of tightly linked mutations, of which only two (*hxn6* and *hxn7*) are presently available, mapped in chromosome VI at about ≈10 cM from mutations in the *hxnS* and *hxnR* genes, which is consistent with the genomic organization described above (J. Kelly & C. Scazzocchio 1984, personal communication).

We isolated from an *A. nidulans* genomic DNA library [23] a plasmid able to complement *hxn6* for growth on 6-NA as sole nitrogen source. The 8256 bp insert comprises *hxnV*, *hxnW*, *hxnX* and partial flanking sequences of the AN9159 and AN9162 loci. The *hxn6* mutation is a G1171A transition within the *hxnV* ORF (see below for correction of the *hxnV* gene model in electronic supplementary material, figure S1) resulting in W296STOP (amber). Southern blots showed *hxn7* to be a chromosomal aberration (possibly an insertion) interrupting the *hxnV* open reading frame (electronic supplementary material, figure S2). The *hxnX* gene (cluster 2/VI) is at 40 748 bps from *hxnZ* (based on genome sequence data [21], while *hxnN* and *hxnM* are adjacent to each other and transcribed from the same strand in chromosome I (cluster 3/I) (figure 1). We obtained cDNAs of all the genes in the three clusters and confirmed that, as gathered by manual inspection and comparative genomics, the database gene models (proposed by automated annotation) for *hxnP*, *hxnZ* and *hxnV* are erroneous (electronic supplementary material, figures S1, S3 and S4 for the correct gene models, table 1 for accession numbers). HxnX, HxnW, HxnV are oxidoreductases, while HxnM and HxnN are hydrolases. A summary of the predicted activities of all the encoded Hxn proteins is shown in table 1.

**Figure 2.**
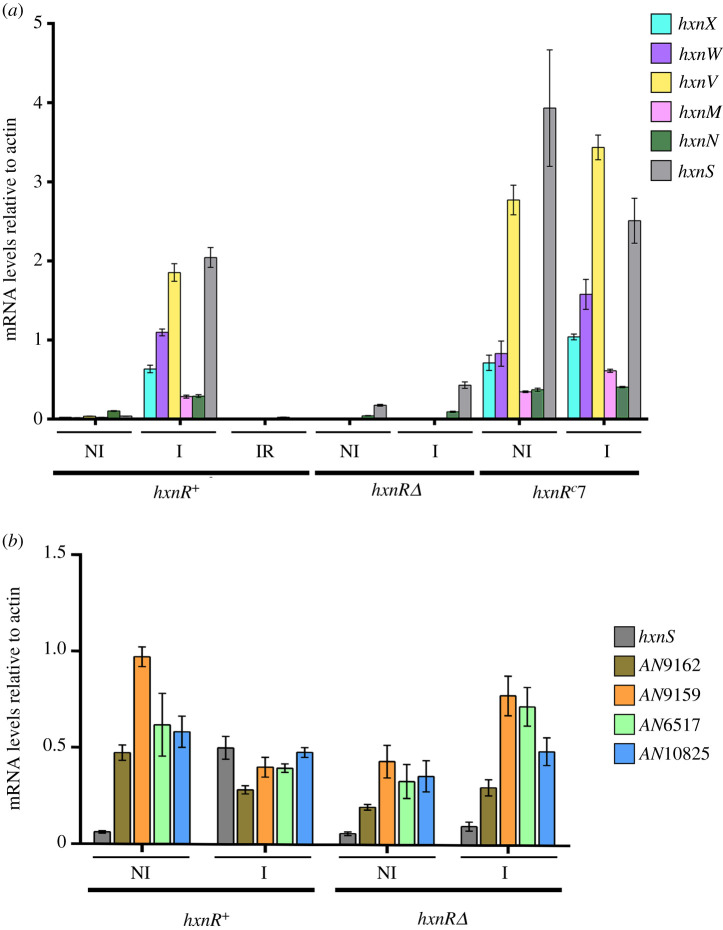
HxnR-dependent co-induction by 6-NA and ammonium repression of genes in clusters 2/VI and 3/I. All genes in clusters 2/VI and 3/I (*a*) and the cognate cluster-flanking genes (*b*) were tested together with *hxnS* (in cluster VI/1), which was included as a positive control of expression. The relative mRNA levels were measured by RT-qPCR and data were processed according to the relative standard curve method [24] with the γ-actin transcript (*actA/*AN6542) as reference. Mycelia were grown on 10 mM acetamide as sole N-source for 8 h at 37°C. They were either kept on the same medium for a further 2 h (non-induced, NI) or induced with 1 mM 6-NA (as the sodium salt, I) or induced as above together with 5 mM of l-(+)di-ammonium-tartrate (induced-repressed, IR), also for 2 h. Strains used were *hxnR^+^* (FGSC A26), *hxnRΔ* (HZS.136) and *hxnR^c^7* (FGSC A872) (electronic supplementary material, table S1). Standard deviations of three independent experiments are shown. Primers are listed in electronic supplementary material, table S2.

**Figure 3.**
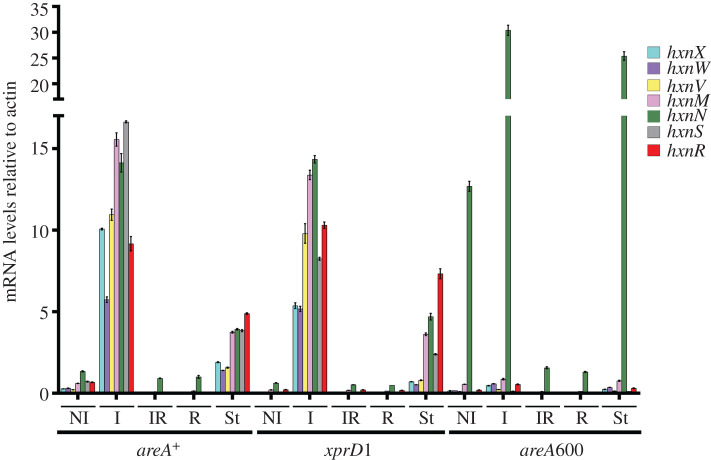
The GATA factor AreA is essential for expression of all *hxn* genes with the exception of *hxnN*. Relative mRNA levels in strains of *areA^+^* (FGSC A26), a generally de-repressed *areA* mutant (*xprD1*, HZS.216) and an *areA*-null mutant (*areA600*, CS3095) were determined (electronic supplementary material, table S1). Non-induced conditions (NI): Strains were grown on MM media with 5 mM l-(+)di-ammonium-tartrate as sole N-source for 8 h, then the mycelia were transferred to MM with 10 mM acetamide for further 2 h. Induced conditions (I): as above but transferred to 10 mM nicotinic acid as sole N-source. Induced-repressed (IR) conditions: transferred to 10 mM nicotinic acid and 5 mM l-(+)di-ammonium-tartrate for further 2 h. N-starvation conditions (St): transferred to nitrogen source-free medium. RT-qPCR data were processed according to the standard curve method [24] with the γ-actin transcript (*actA/*AN6542) as reference. Standard deviations based on three biological replicates are shown. Primers are listed in electronic supplementary material, table S2.

**Figure 4.**
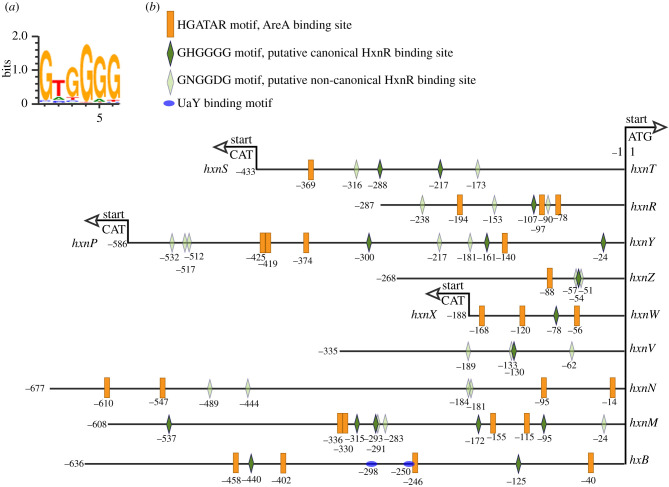
AreA and putative HxnR-binding sites are extant in the 11 genes of the *hxn* regulon. (*a*) Sequence logo of the DNA-binding motif of the HxnR transcription factor generated by the ‘DNA-binding site predictor for Cys2His2 Zinc Finger Proteins' application (http://zf.princeton.edu/) [33]. (*b*) Distribution of 5′HGATAR AreA-binding sites (orange boxes) [32] and putative canonical 5′GHGGGG HxnR-binding sites (dark green lozenges) in *hxn* gene promoters and also in the promoter of the *hxB* gene. The latter encodes a trans-sulphurylase necessary for the activity of the MOCO cofactor in enzymes of the xanthine oxidoreductase group (including HxnS and HxA). UaY-binding sites on the *hxB* promoter are marked by blue coloured ovals [34]. Sequences conforming to the consensus 5′GHGGGG sequence are present in all HxnR-regulated genes, except *hxnN*. Nevertheless, figure 2 shows clearly that *hxnN* is under the control of HxnR. Thus, the physiological binding sites may have a more relaxed consensus sequence. We propose 5′GNGGDG motif as a non-canonical consensus binding site that can be found in *hxnN* as well as in other *hxn* promoters. Light green lozenges indicate the location of the more relaxed consensus 5′GNGGDG motif. Note that the *hxnT*/*hxnS*, *hxnP*/*hxnY* and *hxnX*/*hxnW* gene couples share bi-directional promoters.

All the genes in clusters 2/VI and 3/I show an HxnR-dependent induction by 6-NA (figure 2*a*). In an *hxnR^c^7* strain, the genes show variable levels of constitutive expression (figure 2*a*), as shown before for cluster 1/VI [11]. The boundaries of the newly detected clusters are defined by the completely different pattern of expression of the flanking genes (loci AN9159 and AN9162 for cluster 2/VI, and loci AN6517 and AN10825 for cluster 3/I; figure 2*b*). As previously shown for the genes in cluster 1/VI, these five newly identified *hxn* genes are strongly ammonium repressible (figure 2*a*) and with one exception (*hxnN*, see below), strictly dependent on the AreA GATA factor, mediating nitrogen metabolite de-repression (figure 3). *xprD1* is usually considered to be the most extreme de-repressed allele of the *areA* regulatory gene [25], however, it did not behave as a de-repressed allele for the expression of any *hxn* gene but rather as a partial loss of function allele for *hxnS* and *hxnP* expression [11] while being variable in its effects on the genes in clusters 2/VI and 3/I (figure 3). Similar behaviour was reported for *ureA* (a urea transporter gene) expression [26], which strongly suggests that the phenotypes resulting from this specific mutation are promoter-dependent. The amidase-encoding *hxnN* gene shows a paradoxical pattern of expression. While it is clearly subject to repression by ammonium, it is drastically over-expressed in *areA600* background under neutral (non-induced, non-repressed conditions, see legend to figure 3), as well as under induced and nitrogen starvation conditions (figure 3). As *areA600* is a null mutation due to a chain termination mutation upstream of the DNA-binding domain [27], we must conclude that AreA acts on *hxnN* as a transcriptional repressor, in contrast to its activator function in almost all genes involved in nitrogen source utilization [15,28]. However, *hxnN* is sensitive to ammonium repression, an apparent paradox, which is most probably due to its dependence on HxnR (as seen in figure 2*a*), whose expression is drastically repressed by ammonium [11] (figures 2 and 3). The sensitivity of *hxnN* expression to liganded HxnR is supported by the strikingly higher expression levels seen for *hxnN* under induced conditions, and this in all three *areA* alleles tested. However, the expression of *hxnR* is undetectable or extremely weak (for example under nitrogen starvation conditions) in an *areA600* background, which may suggest that the high expression seen for *hxnN* under those latter conditions is not HxnR dependent. In the absence of evidence for other transcription factors besides HxnR, the repressing effect of AreA seems to affect the basal transcription level of *hxnN* (see below). This contrasts with what was reported for the genes in cluster 1/VI [11]. Other instances of AreA acting as a repressor have been reported, notably *nadA* (encoding adenine deaminase, where induction by ammonium was seen [29]) and the arginine catabolic genes *agaA* (arginase) and *otaA* (ornithine transcarbamylase) [30,31]. We searched the genes in the three clusters for the consensus AreA 5′HGATAR DNA-binding sites [32] (figure 4). The *hxnV* gene upstream sequence does not feature canonical AreA sites; nevertheless, its expression is repressible by ammonium, probably due to indirect repression via repression of *hxnR* transcription. The *hxnR* upstream region shows both canonical AreA sites and one putative HxnR-binding site (see below). This is consistent with this gene being inducible, self-regulated and subject to nitrogen metabolite repression [11] (figure 2). The negative effect of AreA on *hxnN* expression may be due to the presence of a canonical GATA-binding motif (5′AGATAA on the non-coding strand at position-14 to -19), interfering with the start or progress of transcription. This is analogous to the situation observed for *nadA*, where there is a likely steric interference of the binding of AreA with that of the specific transcription factor UaY, the two sites being separated by 3 bp [29].

The binding sites of HxnR have not been experimentally determined, however, they could be predicted with reasonable probability [33]. Besides the consensus 5′HGATAR AreA-binding sites, figure 4 shows also the distribution of the putative canonical and non-canonical HxnR-binding sites (5′GHGGGG and 5′GNGGDG, respectively) in all 11 *hxn* genes as well as in the *hxB* gene (AN1637), encoding a MOCO sulphurylase ([34] for review) necessary for the enzymatic activity of both HxA and HxnS [35]. Two putative canonical HxnR-binding sites are extant in the *hxB* promoter (figure 4). This gene is under the independent and additive control of UaY (the transcription factor regulating the purine utilization pathway) and HxnR [35].

### 2.2. Chromosome rearrangements led to separation of clusters 1/VI and 2/VI in *Aspergillus nidulans* and other *Aspergillus* species

**Figure 5.**
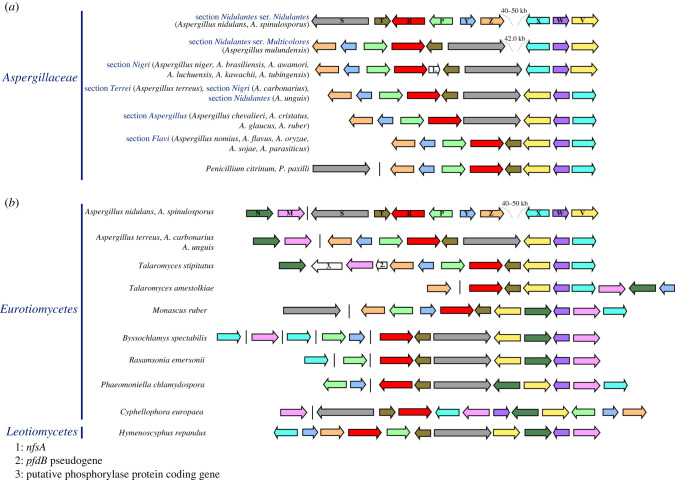
Genomic arrangement of the *hxn* gene clusters. (*a*) Selected species from *Aspergillaceae* including section *Nidulantes*, section *Nigri*, section *Terrei*, section *Aspergillus*, section *Flavi* and *Penicillium* (*b*) selected species from other *Eurotiomycetes* compared with *H. repandus* (*Leotiomycetes*, *Helotiales*). Orthologues found in different species are indicated by arrows of the same colour as in figure 1. A single vertical line symbolizes physical separation of genes on different contigs.

The organization described above and in figure 1 for *A. terreus* (section *Terrei*) is most probably ancestral to *Aspergillus*, as is it seen in species belonging to diverging sections of this genus, namely in *Aspergillus carbonarius* (section *Nigri*) and in *Aspergillus unguis*, an early diverging species of section *Nidulantes* (figure 5*a*). This organization closely resembles the one seen in species of the basal section *Aspergillus* (*A. chevalieri*, *A. cristatus*, *A. glaucus* and *A. ruber*), where, however, the *hxnT* gene is absent (figure 5*a*). Within section *Nidulantes*, the first inversion on chromosome VI resulted in the separation of the clusters 1/VI and 2/VI, inverting the position and orientation of *hxnX*, *hxnW* and *hxnV* (in cluster 2/VI) in relation to *hxnS* (in cluster 1/VI) (figure 5*a*). This results in a gap of 41 992 bp between the two clusters in * Aspergillus mulundensis* (section *Nidulantes*, series *Multicolores* [36]; figure 5*a*). A second inversion within section *Nidulantes* led to the configuration seen in *A. nidulans* and its closest sequenced relative *A. spinulosporus* (section *Nidulantes*, series *Nidulantes*) [36] leaving, respectively, gaps of 40 876 bp and 49 972 bp between *hxnZ* and *hxnX* (figure 5*a*)*. Aspergillus sydowii* and *Aspergillus versicolor* (section *Nidulantes*, series *Versicolores*) [36], also show separation of clusters 1/VI and 2/VI (electronic supplementary material, figure S5A), however, the relative gene orientation and phylogenetic position of these two species strongly suggest that their cluster organization arose from events independent to those described above for *A. nidulans* and *A. spinulosporus* (figure 5*a*). Two distinct independent chromosome inversions, like the one described above for *A. mulundensis* must have occurred within section *Nigri*, leading to the organization seen in *A. aculeatus* and the *A. niger* clade (electronic supplementary material, figure S5A); in *A*. *niger* and allied species, *hxnS* and *hxnX* are abutting neighbours; in *A. aculeatus* (also in section *Nigri*), where *hxnT* is absent there is an approximately 32 kb gap between these genes.

In two species (*Aspergillus steynii* and *Aspergillus westerdijkiae*) of two closely related series (ser. *Steyniorum* and ser. *Circumdati*, respectively), clusters 1 and 2 are separated without any relative change of gene orientation (electronic supplementary material, figure S5A). This could be formally described as an insertion, however, partial DNA identity and gene colinearity in the inter-cluster sequence rather suggest two successive inversions. In *Aspergillus wentii* (section *Cremei*) a rearrangement associated with the loss of *hxnS* separates from the original cluster, a sub-cluster including *hxnZ*, *hxnY* and a pseudogenized *hxnP*; while *hxnV*, *hxnW* and *hxnX* are still included in the main cluster together with the neighbouring *hxnT* and *hxnR* (electronic supplementary material, figure S5A).

### 2.3. In the *Pezizomycotina*, with the exception of *Aspergillus*, the *hxnN* and *hxnM* genes are included in the *hxn* cluster

The enzymes encoded in clusters 1/VI and 2/VI are all oxidoreductase enzymes, however, to release ammonium from NA-derived metabolites, hydrolytic enzymes are necessary [2]. Within the putative *hxn* clusters of many *Pezizomycotina* species, two genes encoding, respectively, a putative cyclic-imide hydrolase (*hxnM* EC 3.5.2.16, greater than 60% identity with AAY98498, the cyclic-imide hydrolase from *Pseudomonas putida* [37]) and a putative amidase (*hxnN* EC 3.5.1.4) are extant. The cognate genes of *A. nidulans* have been described above. In *C. europaea*, *hxnN* and *hxnM* lie in between *hxnX* and *hxnV*, and are separated by *hxnW* constituting two neighbouring, divergently transcribed gene couples, *hxnV*–*hxnN* and *hxnW–hxnM* within the cluster (figure 1). It should be stressed that these two divergently transcribed couples are conserved across different classes of the *Pezizomycotina*, but not in the genus *Aspergillus*, where, with the exception of section *Flavi*, *hxnN* and *hxnM* are separated from the main cluster (electronic supplementary material, figures S5A and S5B).

In *Monascus ruber* (*Eurotiomycetes*, *Eurotiales*, *Aspergillaceae*—same family as *Aspergillus*), where *hxnS* is not included in a 10-member gene cluster (see below), the two divergently transcribed couples are conserved (figure 5*b*). In *Talaromyces* sp. (*Eurotiomycetes*, *Eurotiales*, *Trichocomaceae*) the *hxnM*, *hxnW*, *hxnV* and *hxnN* genes are not arranged in divergent couples (electronic supplementary material, figure S5A). Electronic supplementary material, figure S5B shows a variety of cluster organizations in species of the *Pezizomycotina* and *Saccharomycotina* subphyla, with the *hxnN* and *hxnM* genes showing different patterns of integration within the *hxn* cluster, yet with a remarkable conservation of the *hxnN–hxnV* and *hxnM–hxnW* divergently transcribed couples in classes of *Pezizomycotina* subphyla (electronic supplementary material, figure S5B).

In the genome of *C. europaea*, besides the divergently transcribed couples mentioned above, two other couples are extant: *hxnS–hxnT* and *hxnP–hxnY* (figure 1). These couples are mostly conserved in the *Pezizomycotina*, irrespective of whether all 11 genes are included in a single cluster (electronic supplementary material, figure S5B). Noticeably, in *A. nidulans*, cluster 1/VI comprises *hxnS–hxnT* and *hxnP–hxnY*. In *Hymenoscyphus repandus* (*Leotiomycetes*, *Helotiales*), similarly to *C. europaea*, all 11 genes are included in a single mega-cluster, albeit in a different arrangement; nevertheless, two divergent couples are conserved (*hxnS–hxnT* and *hxnM–hxnW*) (figure 5*b*). A similar conservation of divergently transcribed genes is seen in other gene clusters, such as the DAL cluster of the *Saccharomycetales*, where the *DAL4–DAL1* pair is conserved between *Saccharomyces cerevisiae* and *Naumovia castellii* in spite of two inversions affecting the budding yeast DAL cluster in chromosome IX [38], and in the biotin biosynthesis cluster of the *Pezizomycotina* (*bioF-bioDA*) [39]. The persistence of these divergently transcribed couples could be due to the fact that they share a bi-directional promoter, as established for *GAL10* and *GAL1* in *S. cerevisiae* ([40,41] and references therein) and for *niiA*–*niaD* in *A. nidulans* [42,43].

### 2.4. Evolution of the *hxn* gene cluster(s) in the *Ascomycetes*

Previous work has shown that HxnS is restricted to the *Pezizomycotina* [11]. It is therefore unlikely that other fungi could hydroxylate NA and thus use it as a nitrogen source. However, it is possible that an *hxnS* gene was incorporated into a pre-existent metabolic pathway, whether catabolic or detoxifying, whether or not organized as a cluster. We thus investigated the presence of putative *hxn* clustered genes throughout the fungal kingdom. No putative *hxn* clusters are present in any early divergent fungal lineages in the *Basidiomycota* or in the *Taphrynomycotina*, except that *hxnT*, *hxnN* and *hxnM* unlinked orthologues are present in the early diverging *Taphrinomycotina*, *Saitoella complicata* (for HxnT and HxnM phylogenies see electronic supplementary material, figures S7 and S9).

Clusters comprising *hxn* genes are present in several scattered species of *Saccharomycotina* (electronic supplementary material, figure S5B); however, not in the *Saccharomycetaceae* and *Debaryomycetaceae* families. All species of *Lipomyces*, an early divergent genus of the *Saccharomycotina*, include divergently transcribed *hxnN* and *hxnM* clustered genes (electronic supplementary material, figure S9). The genomes of fourteen scattered species of *Saccharomycotina* (electronic supplementary material, figure S5B) comprise clusters with the *hxn* gene complement, always including the transcription factor *hxnR* and never including *hxnS, hxnZ* and *hxnN*, even if the latter gene could be found unlinked to the cluster in an early divergent species (*Trigonopsis variabilis*)*.* A phylogeny of *hxnR* is shown in electronic supplementary material, figure S6 and is consistent with a monophyletic origin of this gene in the *Saccharomycotina* and *Pezizomycotina*. It seems most unlikely that the clusters of the *Saccharomycotina* have a single origin. The *Lipomyces hxnM*–*hxnN* gene pair is found only in this genus where all other *hxn* genes are absent. Among other families, the occurrence of clusters with variable organizations does not follow any obvious evolutionary pattern. In the fourteen species of *Saccharomycotina* where we found an *hxn* cluster, the *hxnT*, *hxnR* and *hxnV* genes are monophyletic (electronic supplementary material, figures S5–S8). Notwithstanding the above, the phylogeny of *hxnM* suggests several different origins of clustered *hxnM*s within the *Saccharomycetales* from an unclustered paralogue, possibly acquired by HGT (see below, and electronic supplementary material, figure S9). One clustering event occurred in the *Phaffomycetaceae*, possibly two in the *Pichiaceae*, while only one species of the CUG-Ala clade, *Pachysolen tannophilus* [44] includes an *hxn* cluster, with an *hxnM* gene. Among the *Pichiaceae*, in the genus *Ogataea*, the monophyletic origin of clustered and unclustered *hxnM* genes is supported by their intron–exon organization.

Several instances of gene loss, gene duplication and cluster reorganization have occurred in the *Pezizomycotina*. In some *Aspergillus* species, *hxnT* (encoding an FMN-dependent oxidoreductase) is missing from the cluster (electronic supplementary material, figure S5A) and indeed from the genome. In many taxa of *Sordariomycetes* duplication of *hxnV* and subsequent loss of the *hxn* cluster genes can be observed, leaving only an *hxnV* copy and *hxnM* (electronic supplementary material, figure S5B).

It is striking that in the *Aspergillus* section *Flavi*, in *Talaromyces* species and in most species of *Penicillium* the *hxnS* gene is absent and the organization of the whole cluster is completely identical in some species of *Talaromyces*, in most of Penicillia and in *Aspergillus* section *Flavi* (electronic supplementary material, figure S5A). This coincidence indicates possible HGTs between these taxa (see below, HGT between *Talaromyces* and *Aspergillus* section *Flavi*). As the transcription factor-encoding gene *hxnR* is conserved, the implication is that these organisms should be able to use 6-NA but not NA.

### 2.5. Insertion of additional genes within the *hxn* clusters

We define as ‘additional genes’ those that appear sporadically within the *hxn* clusters of some taxa. While we have not investigated the function(s) of these genes, none are extant in the three co-inducible *hxn* clusters of *A. nidulans*. The insertion of a gene encoding a nitro reductase (*nfsA*) originally horizontally transmitted from a cyanobacterium has been discussed previously [11]; the insertion occurred after the divergence of *A. carbonarius* from other members of section *Nigri* [45] (figure 5*a*). The expression of *nfsA* from *A. nidulans* (AN8360) is not regulated by nicotinate or the transcription factor HxnR, strongly suggesting that the gene product is not necessary for nicotinate utilization as a nitrogen source [11].

In the *hxn* cluster of *Aspergillu*s section *Flavi*, and in a number of *Penicillium* and *Talaromyces* species (electronic supplementary material, figures S5, S10 and S11), a gene of unknown function, to be called *pfdB*, for putative *p*eroxisomal *F*MN-dependent *d*ehydrogenase (see below) lies between *hxnZ* and *hxnM*. This is a paralogue of *pfdA*, a gene universally present in the *Pezizomycotina*, which is never included in an *hxn* cluster. Since *pfdB* is not extant in *A. nidulans*, we can exclude that PfdB is necessary for NA utilization as nitrogen source. The encoded Pfd proteins include PF01070.18 (FMN-dependent dehydrogenase) and PF00173.28 (cytochrome b5-like-binding domain) domains and have a canonical PTS1 (peroxisomal entry signal) [46]. The phylogeny of PfdA and PfdB clearly supports a scenario of gene duplication of *pfdA* in the ancestor of Penicillia with simultaneous or subsequent cluster integration (mean similarity between PfdA and PfdB paralogue proteins is 65% compared with 88% of PfdA orthologues among themselves; electronic supplementary material, figure S11). PfdA has a second, distinct paralogue, PfdC, too, which however lost the PTS1 signal in some cases and is only present in section *Flavi*, and in a number of *Talaromyces* and *Penicillium* species and in a few species of other clades (electronic supplementary material, figures S10 and S11). The occurrence of *pfdC* in taxa is consistent with the duplication of the *pfdA* ancestor in an early diverging species followed by several episodes of loss completely unrelated to the evolution of the *hxn* cluster.

In *Penicillium paxilli*, *P. citrinum* and *P. steckii*, a gene encoding a protein of 467–469 residues, comprising a PF00781.24, diacylglycerol kinase catalytic domain, (orthologues annotated as sphingoid long chain kinases) lies between the *hxnZ* and *hxnM* genes. This gene is duplication of a gene present elsewhere in these organisms and omnipresent in the *Eurotiomycetes*. In *Talaromyces stipitatus* a *pfdB* pseudogene is extant between *hxnZ* and *hxnM*, and additionally, an intron-less gene encoding 751 residue-multidomain protein, comprising an N-terminal PF0104820.11 (phosphorylase superfamily N-terminal, most similar to nucleoside phosphorylases) domain and a C-terminal PF05960.11 (bacterial protein of unknown function) domain is located between *hxnN* and *hxnM*, the nearest homologues of the inserted gene being present and unlinked to any *hxn* gene in *Talaromyces verruculosus*.

In *Kregervanrija fluxuum* (*Saccahromycotina*, *Pichiaceae*) a putative amidase gene is inserted in the cluster between *hxnM* and *hxnT* (electronic supplementary material, figure S5B). The encoded protein has only 35% identity with HxnN of *A. nidulans*, compared with the 51% identity shown by the genuine HxnN proteins of *Lipomyces starkeyi*, *T. variabilis* and *S. complicata*. Its nearest homologue is a putative amidase from *Ogataea parapolymorpha* (56% identity). It is tempting to speculate that this amidase has been recruited to the cluster to carry out a similar catalytical function to that afforded by HxnN.

### 2.6. HGT events involving *hxn* genes

The organization of the *hxn* clusters, together with phylogenies of individual genes suggested several episodes of HGT involving individual genes, or in a specific case the whole cluster. These events are discussed below.

### 2.7. HGT of *hxnS*

**Figure 6.**
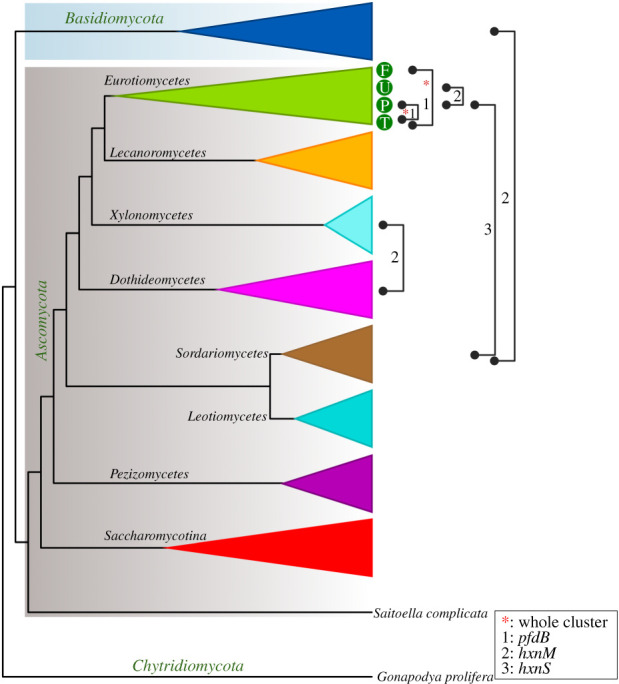
Summary of proposed *hxn* HGT events between fungal taxa. F: *Aspergillus* section *Flavi*; U: *Aspergillus* section *Usti*; P: Penicillia; T: early diverging species of *Talaromyces*; 1: HGT of *pfdB* gene found between Penicillia and species of *Talaromyces* and between species of *Talaromyces* and *Aspergillus* section *Flavi*; 2: HGTs of *hxnM* gene from *Xylonomycetes* (*Symbiotaphrina*) to *Dothideomycetes*; from the common ancestor of Fusaria (*Ascomycota*, *Sordariomycetes*) to *Basidiomycota* (the common ancestor of the *Panellus stipticus* and *Mycena galopus* belonging to *Agaricomycetes*) and from Penicillia (*Ascomycota*, *Eurotiomycetes*) to *Aspergillus* section *Usti* (*Ascomycota*, *Eurotiomycetes*) (see detailed phylogeny in electronic supplementary material, figure S9); 3: HGT of *hxnS* gene from *Aspergillus* section Usti to *Penicillium* section *Citrina*; red asterisk: transfer of the whole *hxn* cluster composed of nine *hxn* genes and including the *pfdB* gene from Penicillia to species of *Talaromyces* and from *Talaromyces* to *Aspergillus* section *Flavi*. Lines connecting taxa mark confirmed HGTs.

In the genome of most *Pezizomycotina*, an *hxnS* gene, encoding the first enzyme of the nicotinate utilization pathway, is extant [11]. However, in most Penicillia and *Talaromyces* species the *hxnS* gene is absent. In some *Talaromyces* species where *hxnS* is extant and it is unclustered with other *hxn* genes, these *hxnS* genes are the closest orthologues of the *hxnS* of *Monascus* species, consistent with standard phylogeny [11] (electronic supplementary material, figure S12). A different situation occurs in some Penicillia, where *hxnS* occurs. The *hxnS* genes from three sister species of section *Citrina*, [47] (*P. citrinum*, *P. paxilli* and *P. steckii*) were reacquired by HGT from either a *Fusarium* or a *Colletotrichum* species (*Sordariomycetes* [11]; figure 6; electronic supplementary material, figure S12).

### 2.8. Possible HGT and clustering events involving the *hxnM* gene

In all investigated dikarya, HxnM paralogues, presumably non-related to NA metabolism, are extant. Based on a comprehensive phylogeny of cluster-related and cluster-non-related HxnM and its paralogues (electronic supplementary material, figure S9) subjected to reconciliation with the species tree (using GeneRax), we confirmed HGTs among *Ascomycota* taxa and HGT from *Ascomycota* to the common ancestor of two species of *Basidiomycota* (summarized in figure 6 with details in the legend, and in electronic supplementary material, figure S9). Since these two *Basidiomycota* species (*Panellus stipticus* and *Mycena galopus*) have only a single, Ascomycota-derived (from common ancestor of Fusaria) *hxnM* gene, the *Basidiomycota hxnM* must necessarily have been lost from an ancestor of these two Basidiomycota species.

Electronic supplementary material, figure S9 is consistent with a vertical inheritance of *hxnM* homologues in the dikarya, excluding a recent HGT from bacteria. The phylogeny of HxnM is compatible with an originally unclustered *hxnM* homologue being duplicated, one copy being recruited in an *hxn* cluster. Details are shown in electronic supplementary material, figure S9 and the cognate legend.

While the clustered *hxnM* genes appear monophyletic, originating from the same clade of unclustered genes, clustering in the *Pezizomycotina* occurred independently from that within the *Saccharomycotina*, followed by several independent instances of separation of an *hxnN*–*hxnM* minicluster (such as detailed above for the *Aspergillus*) and presence of an *hxnM* unclustered homologue, as it occurred in the *Leotiomycetes*.

The clade comprising the HxnM homologues of the *Saccharomycotina* seems monophyletic (electronic supplementary material, figure S9). However, it does not occur as expected as a sister clade of all the homologues of the *Pezizomycotina*, but within the different *Pezizomycotina* clades. The low aLRT value at the relevant node, however, neither supports nor excludes *Saccharomycotina* acquiring an *hxnM* gene by HGT from *Pezizomycotina* (electronic supplementary material, figure S9).

### 2.9. HGT events of whole *hxn* clusters

**Figure 7.**
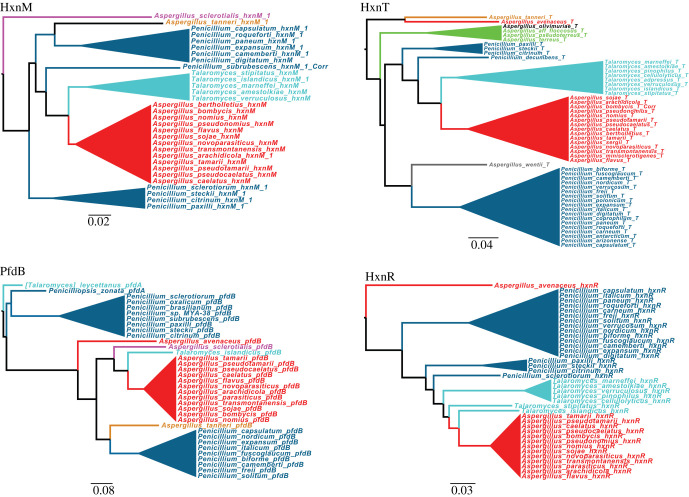
HGT from *Talaromyces* to *Aspergillus* section *Flavi* is supported by the phylogenies of four different proteins based on *Eurotiomycetes* data set (see electronic supplementary material, figures S6, S7, S9 and S10 for the complete phylogenies). Cyan: *Talaromyces*; blue: *Penicillium*; red: *Aspergillus* section *Flavi*; purple: *Aspergillus* section *Polypaecilum*; light brown: *Aspergillus* section *Tannerorum*; green: *Aspergillus* section *Terrei*; grey: *Aspergillus* section *Cremei*; black: *Aspergillus* section *Flavipedes*. When *hxnM* paralogues are extant in the genome, the cluster-related protein is called HxnM1, while the cluster-non-related paralogues are numbered consecutively. Otherwise, when no paralogues are extant in the genome, the cluster-related protein is referred to as HxnM.

Reconciliation of the phylogeny of PfdBs extant in *Eurotiomycetes* with the species tree (by using GeneRax) confirmed that the *pfdB* of *Talaromyces* which was acquired by HGT from an ancestral species of Penicillia was further transferred from a *Talaromyces* by HGT (together with the whole *hxn* cluster) to an ancestor of *Aspergillus* section *Flavi* (figure 7). Since Penicillia and *Aspergillus* section *Flavi* share an identical cluster organization with some species of *Talaromyces*, the HGT events most probably involved two episodes of HGT of the whole *hxn* cluster (figure 6). This outlines a scenario by which, after the appearance of *pfdB* by a single gene duplication of *pfdA* in the ancestral species of Penicillia, *pfdB* subsequently integrated into the cluster in this genus. An HGT of the whole cluster to an early diverging species of *Talaromyces* would have occurred followed by a further HGT from *Talaromyces* to the ancestor of *Aspergillus* section *Flavi*. This scenario implies that the putative acceptor ancestor *Aspergillus* of section *Flavi* must have lost previously the cluster present in other *Aspergillus* species. This is strikingly confirmed by genomes of early diverging species of section *Flavi* (*A. leporis*, *A. alliaceus* and *A. bertholletius*), which show both instances of *hxn* gene loss and presence of *hxn* pseudogenes (electronic supplementary material, figure S5A). The most extreme case being that of *A. coremiiformis*, where no *hxn* genes are present. In *A. bertholletius* a cluster of 7 *hxn* pseudogenes is extant, where the only intact gene is *hxnT* (electronic supplementary material, figure S5A), however this gene is not a fossil, but it derives from *Talaromyces* by HGT (electronic supplementary material, figure S7). The earliest diverged species of section *Flavi* is supposed to be *A. avenaceus* [48,49]. This is fully supported by the position of the cluster-independent *pfdA* and *pfdC* genes in the phylogenetic tree (electronic supplementary material, figure S10). The cluster of this species, which includes *pfdB*, is similar to that of other *Flavi*, except that *hxnP* is missing and neither of the two *hxnM* paralogues is included in the cluster.

The phylogenies of HxnR, HxnT and HxnM are consistent with the HGT scenario described above for *pfdB*, however reconciliation analysis restricted to phylogenies of the *Eurotiomycetes* confirmed the proposed HGT event only for HxnR (figure 7). In spite of this apparent contradiction, the evidence strongly suggests the whole HG transfer of the cluster as detailed above (figure 7).

Disturbingly, in the *hxnR*, *hxnV* and *hxnT* phylogenies, *A. avenaceus* appears as out-species of the *Talaromyces*/*Penicillium* clade which transferred the cluster to other *Flavi* (figure 7; electronic supplementary material, figures S6–S8). There is obviously a complex series of HGTs which may be solved when more genomes of closely related species become available.

A number of *Aspergillus* species have undergone episodes of HGT, gene loss and even whole cluster duplication. These events are described in electronic supplementary material, figure S5.

### 2.10. Concluding remarks

Experimental work has shown that three gene clusters in *A. nidulans* constitute a nicotinate (actually a nicotinate derivative) inducible regulon, under the control of a specific Zn-finger transcription factor, HxnR. Deletion of HxnR has shown that expression of some or all of the genes in this regulon are necessary for NA, 6-NA and the putative intermediate 2,5-DP utilization as nitrogen sources [11]. Our previous results [11] show that at least the latter compound is toxic. This may be relevant when discussing the hypothesis that clustering is evolutionary favoured in pathways where such toxic intermediated are extant [20]. The specific metabolic function of each encoded protein will be reported separately, together with the identification of intermediate metabolites, including additional toxic ones. The *hxn* regulon is extant only in the *Ascomycetes*, the variable organization seen in different species includes instances of complete clustering of all 11 genes, which may suggest an evolutionary pressure towards the integration of the whole *hxn* gene complement. However, instances of declustering such as the separation of clusters 1/VI and 2/VI in section *Nidulantes* of *Aspergillus* occurred. Different cluster arrangements may have different adaptive values in organisms with different ecologies and physiologies. Rearrangements might be accounted for aleatory recombinational events with no obvious selective aftermath. The *hxn* cluster may alternatively or additionally be a hot spot of recombination. Several instances of HGT were detected (figure 6), most notably the origin of the cluster of *Aspergillus* section *Flavi* from *Talaromyces*/Penicillia. The events of HGT, together with the recruitment of genes after duplication, including *hxnS* and *hxnM*, and additional genes such as *pfdB*, underlie both the dynamic nature and the reticulate character of metabolic cluster evolution, thus providing a perhaps unique window on the evolutionary events underlying cluster organization plasticity.

## 3. Material and methods

### 3.1. Strains and growth conditions

The *A. nidulans* strains used in this work are listed in electronic supplementary material, table S1. Standard genetic markers are described in http://www.fgsc.net/Aspergillus/gene_list/. Minimal media (MM) contained glucose as the carbon source; the nitrogen source varied according to the experimental condition [11]. The media were supplemented according to the requirements of each auxotrophic strain (www.fgsc.net). Nitrogen sources, inducers and repressors were used at the following concentrations: 10 mM acetamide, 10 mM NA (1 : 100 dilution from 1 M NA dissolved in 1 M sodium hydroxide) and 5 mM l-(+)di-ammonium-tartrate as sole N-sources; 1 mM 6-NA sodium salt as inducer and 5 mM l-(+)di-ammonium-tartrate as repressor. Growth conditions are detailed in the figure legends of corresponding experiments.

### 3.2. RNA manipulation

Total RNA was isolated using a NucleoSpin RNA Plant Kit (Macherey-Nagel) and RNase-Free DNase (Qiagen) according to the manufacturer's instructions. cDNA synthesis was carried out with a mixture of oligo-dT and random primers using a RevertAid First Strand cDNA Synthesis Kit (Fermentas). Quantitative RT-PCR (RT-qPCR) were carried out in a CFX96 Real Time PCR System (BioRad) with SYBR Green/Fluorescein qPCR Master Mix (Fermentas) reaction mixture (94°C 3 min followed by 40 cycles of 94°C 15 s and 60°C 1 min). Data processing was done by the standard curve method [24]. DNA sequencing was done by the Sanger sequencing service of LGC (http://www.lgcgroup.com). Primers used are listed in electronic supplementary material, table S2.

### 3.3. Data mining

The coding sequences of fungal *hxn* genes (ATG-STOP) were mined by TBLASTN screening of DNA databases at the NCBI servers, mainly the Whole Genome Shotgun contigs (WGS) database, using the available online tools [50]. For a few species (*Neurospora crassa*, *Podospora anserina*, *Penicillium chrysogenum*, *Aspergillus oryzae*, *A. niger* ATCC 1015, *Leptosphaeria maculans* and some *Saccharomycotina*), the sequence contings of the published genome are located in the nr/nt database or the Refseq genome database. Additional *Eurotiales* genomes (outside *Aspergillaceae*) are publicly accessible at the website of the Centre for Structural and Functional Genomics (Concordia University Montreal, Canada; https://gb.fungalgenomics.ca/portal/). We also included some species from the 1000 Fungal Genomes project (http://1000.fungalgenomes.org) exclusively available at the Mycocosm database (Joint Genome Institute, US Department of Energy) (https://mycocosm.jgi.doe.gov/mycocosm/home). For the two classes of *Pezizomycotina* for which few genome sequences are public (*Xylonomycetes*, *Pezizomycetes*), we have obtained permission to use the *hxn* complement in the genome sequences of five species lodged at JGI in our current work: *Symbiotaphrina kochii* (project ID: 404190); *Trinosporium guianense* (project ID: 1040180); *Gyromitra esculenta* (project ID: 1051239); *Plectania melastoma* (project ID: 1040543); and *Sarcoscypha coccinea* (project ID: 1042915). TBLASTN query sequences for the 11 *hxn* genes were the full-length proteins deduced from the cDNA sequences we experimentally determined for each of the *A. nidulans hxn* genes (see table 1 for GenBank Accession numbers). Where necessary, to confirm gene orthology among multiple homologous sequences, the TBLASTN hits and their surrounding sequences were further inspected for the conservation of occupied intron positions between species and for colinearity with other *hxn* genes in the sequence contig identified (gene clustering). We did not use the results of automated annotation (‘Models’ or ‘mRNA’ at nr/nt) nor did we use deduced protein databases for the eukaryotic (Hxn) proteins. We used a selection of autoannotated proteins for the prokaryote HxnM outgroup extracted from the nr/nt database, using the *P. putida* cyclic-imide hydrolase (GenBank AAY98498 [37]) as the BLASTP query. We manually predicted the intron–exon structure of each (*hxn*) gene, guided by comparative genomics and after (*in silico*) intron removal deduced the encoded proteins subsequently used in phylogenetic analyses (see below). Alternative yeast nuclear codes were used where appropriate (*Pachysolen*: CUG = Ala, *Priceomyces*: CUG = Ser). For some species in under-represented taxa, we could use the transcriptome shotgun assembly database to obtain intron-less sequences coding for full-length protein.

### 3.4. Construction of maximum-likelihood trees

Criteria for identification of orthologues/paralogues are detailed for each tree. Alignments were done with MAFFT G-INS-i unless otherwise indicated, with default parameters [51,52] (https://mafft.cbrc.jp/alignment/server/). Alignments were trimmed with BMGE with default parameters unless otherwise indicated (https://ngphylogeny.fr/workflows/wkmake/42f42d079b0a46e9, [53]. Maximum-likelihood trees were constructed with PhyML 3.0 using LG model with gamma rate heterogeneity. Automatic model selection was done by SMS (http://www.atgc-montpellier.fr/phyml [54,55]) and the best ML trees were drawn with FigTree v. 1.4.4. Values at nodes of all trees are aLRTs (approximate-likelihood ratio test [56]). All trees are shown in a circular cartooned form. Trees are rooted in the specified out group. Reconciliation was done by GeneRax v. 1.2.3, a maximum-likelihood-based method [57] with default settings using the LG evolutionary model with gamma rate heterogeneity in 500 replicates. Only those transfers were considered, which were present in at least 70% of the replicates. Species tree for the reconciliation was drawn after [58,59].

## Data accessibility

The datasets supporting this article are included in the paper and detailed in electronic supplementary material, tables. Sequences determined by us are available under GenBank accession nos. MT707473, MT707472, MN718567, MN718568, MN718569, MN718566, MN718565, KX585439, MT707474, MT707475. The data are provided in electronic supplementary material [60].
